# Cultural Influences on Dietary Self-Management of Type 2 Diabetes in East Asian Americans: A Mixed-Methods Systematic Review

**DOI:** 10.1089/heq.2019.0087

**Published:** 2020-03-18

**Authors:** Tony Li-Geng, Jessica Kilham, Katherine M. McLeod

**Affiliations:** ^1^ Frank H. Netter MD School of Medicine, Quinnipiac University, North Haven, Connecticut.; ^2^ University of Massachusetts Medical School, Worcester, Massachusetts.

**Keywords:** diabetes, Asian American, self-management, diet

## Abstract

**Purpose:** Many East Asian Americans (EAAs) (populations originating from China, Korea, Japan, and Taiwan) with type 2 diabetes mellitus (T2DM) experience unique challenges in managing their disease, including language barriers and traditional cultural beliefs, particularly among first-generation immigrants.. The purpose of this mixed-methods systematic review was to examine cultural perspectives of EAAs that influence dietary self-management of T2DM and identify education interventions and their approaches to enhance EAAs' dietary self-management of diabetes.

**Methods:** A mixed-methods systematic review was conducted to examine EAAs' perspectives from qualitative studies and to identify education interventions and their approaches from quantitative studies. A literature search was conducted using PubMed/MEDLINE, SCOPUS, CINAHL, and Web of Science from 1995 to 2018. Sixteen studies (10 qualitative and 6 quantitative) met criteria for analysis. Thematic synthesis of qualitative data was conducted using a line-by-line coding strategy. Extracted quantitative data were assessed for cultural approaches used in the interventions and diabetes-related outcomes.

**Results:** In the qualitative studies, beliefs about food impacted EAAs' abilities to adopt appropriate dietary recommendations for diabetes management. Requiring a specialized diet disrupted social harmony and made EAAs feel burdensome to others. Having bilingual and bicultural resources eased the stress of making dietary modifications. The most commonly incorporated approaches in diabetes education interventions were bilingual education and culturally specific dietary recommendations. Social roles and harmony were not discussed. Significant reductions in hemoglobin A1c and increases in diabetes knowledge were reported post-intervention.

**Conclusions:** Beliefs about food, beliefs about social roles, and access to culturally competent care play an important role in dietary self-management of T2DM among EAAs. Understanding the cultural influences on dietary self-management of T2DM and tailoring interventions to meet the needs of EAAs are essential in effort to address the growing epidemic and improve patient outcomes.

## Introduction

Type 2 diabetes mellitus (T2DM) is a growing epidemic among the Asian American population, who also represent the fastest growing immigrant population in the United States.^1,2^ Compared to non-Hispanic whites, Asian Americans have increasingly elevated prevalence rates of T2DM and are 10% more likely to be diagnosed with diabetes than non-Hispanic whites.^3,4^ Data from the 2011–2012 National Health and Nutrition Examination Survey (NHANES)^5^ indicate that 20.6% of Asian Americans have diabetes, 32.2% have pre-diabetes, and 50.9% of cases go undiagnosed, higher than all other racial or ethnic groups.

Notably, the prevalence of T2DM varies significantly across Asian American ethnic groups with consistent evidence indicating that South Asian, Filipino, Pacific Islander, and Japanese groups have the highest prevalence of T2DM.^5,6^ Presently, East Asians (populations originating from China, Korea, Japan, and Taiwan) make up the largest Asian American subpopulation (8.2 million), and this number continues to surge.^4^ While T2DM is less prevalent among the East Asian subpopulation compared to the South Asian and Pacific Islander American populations, the disease remains largely underdiagnosed and undermanaged, highlighting the need for culturally tailored prevention efforts to meet the unique needs of the various Asian American ethnic groups.^5,7^

East Asian Americans (EAAs) are often first-generation immigrants with unique needs in self-management of the disease, especially those related to cultural competency.^8^ Several barriers that influence poor T2DM self-management among EAAs have been identified, including traditional cultural beliefs and norms, language barriers, low health literacy, access to care, social support networks, and diet.^8–12^ In fact, more than half of EAAs report limited English proficiency^13^ and difficulty communicating with their health care providers.^14^ Dietary self-management of T2DM is critical in the treatment of T2DM,^15,16^ yet the traditional East Asian diet is high in refined carbohydrates, including white rice and other refined grains, which are linked to increased risk of diabetes.^17^ In addition, dietary acculturation, specifically the adoption of American dietary habits, is associated with suboptimal dietary choices (e.g., convenience food) and patterns (e.g., large portion sizes)^18^ that hinder the management T2DM.^19,20^

With the rapidly growing Asian American population and increasing prevalence of T2DM, attention has been directed to the prevention and self-management of T2DM among Asian Americans, including EAAs, with the development of bilingual and culturally tailored education interventions.^21,22^ Diet modulation is particularly important to self-management of T2DM; however, less is known about the cultural perspectives, including beliefs and barriers, which influence dietary self-management among EAAs. A number of qualitative studies have helped outline some of the barriers that patients face, but a better understanding of the specific strategies addressed in the development and implementation of culturally tailored interventions is needed, particularly as it relates to dietary self-management of T2DM.

The purpose of this mixed-methods systematic review was to (1) examine the cultural perspectives of EAAs that influence dietary self-management of T2DM and (2) identify culturally tailored education interventions and highlight the approaches used to enhance dietary self-management of T2DM in EAAs.

## Methods

### Study design

A mixed-methods systematic review design was used to integrate findings from qualitative and quantitative studies. This approach allowed for the synthesis of multiple forms of data to provide a rich and detailed understanding of cultural influences, as well as to better direct practice and future research.^23^ The review of qualitative findings allowed for thematic synthesis of EAAs' perspectives and experiences with dietary self-management of diabetes, while the review of quantitative findings allowed for synthesis of data from education interventions and their approaches to influence dietary self-management of diabetes. The review was conducted in accordance with the PRISMA (Preferred Reporting Items for Systematic Review and Meta-Analysis) checklist. Internal Review Board approval was not required.

### Search strategy

Electronic searches of peer-reviewed journal articles were conducted by a medical librarian in the databases PubMed/MEDLINE, SCOPUS, CINAHL, and Web of Science. The PICOS framework was used to develop the search strategy and the following medical subject headings and free text terms were searched: “Asian Americans” or “Chinese American” or “Korean American” or “Japanese American” or “Emigrants and Immigrants” and “acculturation” or “cultural assimilation” or “sociological factors” or “western diet” and “diabetes mellitus” or “type 2 diabetes mellitus” or “glucose intolerance.” Included studies met criteria of (1) EAAs ≥18 years of age as participants, (2) assessing dietary management of T2DM, and (3) employing a qualitative study design, mixed-methods design, or experimental design. Review articles were excluded. The search was limited to studies published in English language from 1995 to 2018.

### Study selection

A total of 688 articles were retrieved in the initial search query. We removed 63 duplicates, resulting in 625 abstracts for screening. To reduce the risk of bias, two reviewers, T.L.-G. and K.M., independently screened the articles in three stages: (1) titles and abstracts for eligible articles; (2) full-text articles for inclusion in the final review; and (3) data extraction for all eligible articles.

**FIG. 1.**
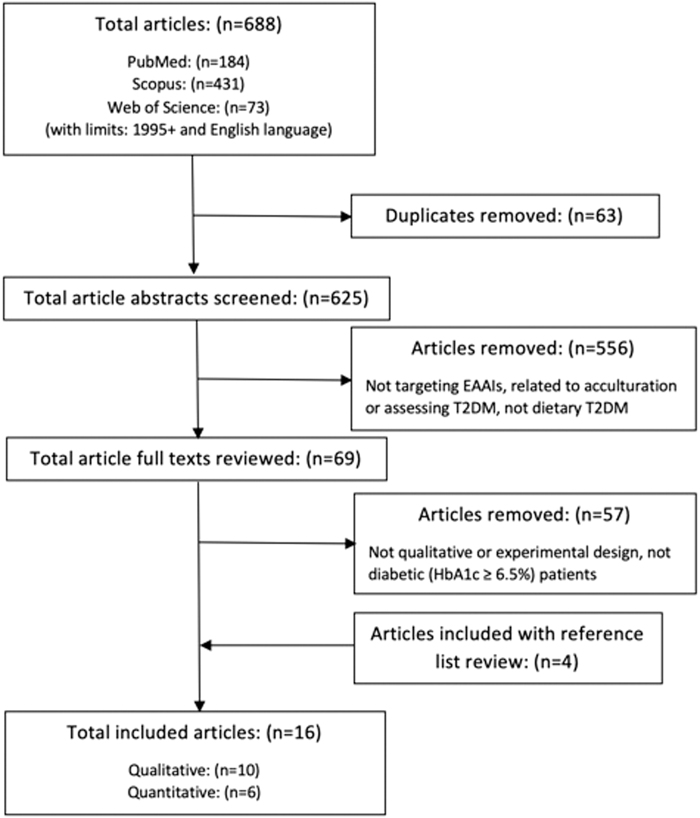
Search strategy for identifying studies on diabetes self-management in EAAs. EAA, East Asian American.

Four articles meeting the inclusion criteria were found by reviewing reference lists of articles that were eligible for inclusion. The level of agreement between the two reviewers was 97.4%. In cases of discrepancies of opinion, the reviewers discussed the article in question and a consensus was reached. Ultimately, 16 articles—10 qualitative and 6 quantitative—were included in the review.^10,24–38^ Figure 1 flowchart illustrates the search process.

### Quality appraisal and data extraction

Pluye's mixed-methods appraisal tool^39^ was used to score and appraise the methodological quality of the final articles retained for the review. The scoring system rates studies based on a set of two screening questions and four specific questions based on qualitative or quantitative study design. Only studies that had a response of “yes” to the following screening questions were included in the review: “Are there clear research questions?” and “Do the collected data allow to address the research questions?” Responses to the specific questions were tallied as “yes” or “no” and percent “yes” response was used as the quality score reported.

All studies had research designs appropriate for their specific aims. Quantitative studies were further subdivided into randomized, nonrandomized, and descriptive categories for appraisal. Of two studies utilizing randomized controlled trials, scores were both 75% because the nature of the study did not allow for blinding.^30,37^ One study with a controlled cohort design scored 100%.^29^ Three studies used a single-group cohort design, of which two scored 100%^34,38^ and one scored 75% due to having less than 80% complete outcomes data.^27^

A data extraction spreadsheet (Table 1) was created to abstract the following data from all 16 articles: authors, year of publication, title, study design, sample size, ethnic self-identity, geographic location, age, baseline hemoglobin A1c (HbA1c) level, stated aim, and key findings. For the 10 qualitative studies, specific data about % women in sample, years lived with diagnosed T2DM, and interviewer description were also extracted. From the 6 quantitative studies, specific data were also extracted about intervention design, cultural approaches used in the intervention, study outcome measures, and major findings.

**Table 1.** Key Characteristics and Findings of Qualitative Studies on Diabetes Self-Management in East Asian Americans

Qualitative studies	Stated aims/focus	Research design	Population	Setting	HbA1c	Interviewer description	Key findings
Chun and Chesla^36^	Explore family members' views and responses to T2DM	Group interview	20 Chinese Americans (35% female), mean age 60.4±8.0 years	Northern California	7.05%±0.98	5 of 11 interviews in English, 6 of 11 interviews in Cantonese by bilingual researchers	Difficulty understanding diabetes as a disease of insulin, belief that illness can be treated with more food, fear of burdening the group
Chesla and Chun^25^	Articulate familial processes of response to T2DM	Small group interview	20 Chinese Americans (35% female), mean age 60.4±8.0 years	Northern California	7.05%±0.98	5 of 11 interviews in English, 6 of 11 interviews in Cantonese by bilingual researchers	Fear of burdening the group, importance of spousal support, view that sweet foods help heal rather than cause disease
Chesla et al.^10^	How cultural and family context make care of T2DM unique or challenging	Couples, group, and individual interview	40 Chinese Americans (60% female), mean age 62±9.2 years	Northern California	6.93%±0.96	All interviews in Cantonese by bilingual researchers	Food restrictions a cause of family conflict, view of rice as a vital food, traditional view of balancing food in conflict with diabetes diet
Chun et al.^28^	Articulate ways that acculturation affects diabetes management	Small group, and individual interview	40 Chinese Americans (60% female), mean age 62±9.2 years	Northern California	6.93%±0.96	All interviews in Cantonese by bilingual researchers	Not have culturally competent medical staff, limited bilingual health education resources
Wang et al.^35^	Understand factors affecting blood glucose control	Focus group interview	24 Chinese Americans (25% female), mean age 59.3 years ±, data not provided	New York City	Data not provided	All interviews in Mandarin by bilingual moderator	Limited bilingual resources, having a positive relationship with health care providers is critical
Cha et al.^24^	Explore views about diabetes management to create culturally specific diabetes program	Individual and couples interview	20 Korean Americans (55% female), mean age 64.5±11.6 years	Atlanta	Data not provided	All interviews in Korean by bilingual researchers	Difficulty with following dietary recommendations due to views on rice and good foods, did not feel understood by physicians
Nam et al.^32^	Examine challenges in diabetes self-management	Small focus group interview	23 Korean Americans (39% female), mean age 58.5±7.3 years	Washington-Baltimore	9.2%±2.8	All interviews in Korean by bilingual researchers	Fear of burdening others with diagnosis, fear of straining spousal relationship with care
Leung et al.^31^	Exploration of reasons why patients have difficulty with health information and communicating needs	Small focus group, individual interview	29 Chinese Americans (38% female), mean age 63.6±12.2 years	Los Angeles	Data not provided	All interviews in Mandarin or Cantonese by bilingual researchers	Reluctance to discuss concerns with physicians, limited bilingual health resources, fear of burdening family with diet
Pistulka et al.^33^	Examine the illness experience of middle-aged Korean Americans living with T2DM and hypertension	Interpretive description, individual interview	12 Korean Americans (67% female), mean age 55.9 ±, data not provided	Baltimore	Data not provided	All interviews in Korean by bilingual researchers	Fear and embarrassment of burdening others with diet
Choi et al.^26^	Understand the characteristics of spousal support for diabetes self-management among Korean diabetics and spouses	Interpretive description, focus group interview	33 Korean Americans (48% female), mean age 71.1±5.9 years	Orange County, California	Data not provided	Interviews in Korean and English by bilingual researchers	Diabetes management negatively affected spousal relationships

HbA1c, hemoglobin A1c; T2DM, type 2 diabetes mellitus.

### Data synthesis and analysis

#### Synthesis of qualitative studies

Thematic synthesis of qualitative data as described by Thomas and Harden^40^ was performed initially by coding quotes and comments using a line-by-line coding strategy from each qualitative study. All codes relevant to dietary self-management of T2DM were extracted and included for thematic synthesis.

Themes that the original study authors noted about the findings were used as a guideline for thematic analysis in this review. Common topics were identified using line-by-line coding. Specifically, codes that indicated a relationship with dietary management were selected for further evaluation.

#### Synthesis of quantitative studies

From the extracted data, relationships between cultural approaches used in the interventions, how study authors defined acculturation, and the relationship to T2DM outcomes were reviewed. Due to the degree of variability in methodological and intervention design of the studies, including differences in education intervention format, frequency, and duration of education sessions, a meta-analysis was not performed. In the studies reporting HbA1c levels, mean decrease in HbA1c levels, Cohen's *d* for effect size, and 95% confidence intervals were calculated and reported.

#### Synthesis of qualitative and quantitative studies

An integrated approach to synthesis of the qualitative and quantitative data was applied. The themes determined in the qualitative synthesis were evaluated in the quantitative studies to determine the extent to which EAAs' perspectives and experiences have been considered or incorporated in the approaches used in education interventions for T2DM self-management.

## Results

### Characteristics of included qualitative studies

Ten qualitative studies were reviewed in total (*n*=261 participants)^10,24–26,28,31–33,35,36^ (Table 1). Two studies used phenomenology to understand the role of family in diabetes self-management, two studies used interpretive comparative methods to compare results between two groups with similar demographics, and six studies used interpretive descriptive methods. Five studies interviewed both T2DM patients and their spouses^10,25,26,28,36^ and five studies interviewed individual patients.^24,31–33,35^

Six studies conducted in Northern California, New York City, and Los Angeles, CA, included participants originating from China. The languages spoken by participants included Cantonese, Mandarin, or English. Four remaining studies were conducted in Baltimore, MD, Washington, DC, Atlanta, GA, and Orange County, CA, and included participants originating from Korea who spoke either Korean or English. No studies of Japanese Americans were identified in the review. The average age of participants ranged from 55 to 68 years and average time lived in the United States ranged from 14 to 25 years. The majority of participants (57%) in the studies was male.

### Thematic synthesis of qualitative review findings

**FIG. 2.**
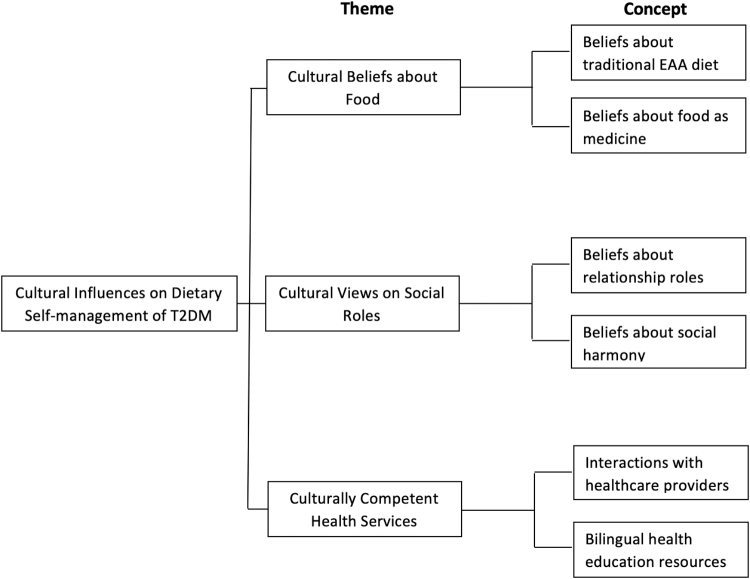
Key concepts synthesized from qualitative studies on diabetes self-management in EAAs.

After compilation of codes and themes (Table 2), data analysis identified three themes characterizing cultural perspectives and experiences that influence dietary self-management of T2DM, including cultural beliefs about food, cultural views on social roles, and culturally competent health services. Analytical themes and concepts are presented in Figure 2.

**Table 2.** Coding of Key Findings of Qualitative Studies on Diabetes Self-Management in East Asian Americans

Article	Sample extracted data	Key concepts
Chesla and Chun^25^	Being discreet about disease to avoid burdening others with dietary restrictions in communal setting	Beliefs about social harmony
	Spousal support critical to disease self-management	Beliefs about relationship roles
	Avoiding foods traditionally thought to heal, like sweets	Beliefs about food as medicine
Chesla et al.^10^	Families challenged by conflicts centered around food restrictions	Beliefs about relationship roles
	Difficulty accommodating new diet—rice is a staple and symbolically vital food	Beliefs about traditional EAA diet
	Food needs to be eaten with a balance between “hot” and “cold” to maintain health	Beliefs about food as medicine
Chun et al.^28^	Having culturally competent medical staff with knowledge of appropriate dietary practices enhances medical practice	Interactions with health care providers
	Bilingual health education materials help provide information	Bilingual health education resources
Wang et al.^35^	Lack of bilingual materials on disease made it harder to manage	Bilingual health education resources
	Having a positive relationship with their PCP was critical	Interactions with health care providers
Cha et al.^24^	Often a struggle to follow dietary recommendations due to traditional views of rice and meat to be good foods	Beliefs about traditional EAA diet, Belief about food as medicine
	Felt disconnected with PCP, did not feel physicians understood their concerns	Interactions with health care providers
Nam et al.^32^	Reluctance to disclose diagnosis to others to avoid being seen as a burden	Beliefs about social harmony
	Dietary self-management puts strains on relationships with spouses	Beliefs about relationship roles
Leung et al.^31^	Reluctant to confront physicians about issues/concerns about management	Interactions with health care providers
	Limited bilingual health information in the community, not well distributed	Bilingual health education
	Avoid burdening others, particularly family, with low-sugar, low-fat diet	Beliefs about social harmony
Chun and Chesla^36^	Difficulty understanding diabetes due to view of diet as a balance of cold and hot foods and not as a disease of insulin	Belief about traditional EAA diet, belief about food as medicine
	Food is an essential ingredient to quality of life, people with illnesses are given food	Belief about food as medicine
	Food restriction conflicts with collectivist norms of prioritizing the group	Beliefs about social harmony
Pistulka et al.^33^	Fear being a burden to others by forcing them to accommodate patients during meals. Feel embarrassed in that situation	Belief about social harmony
Choi et al.^26^	Conflicts about dietary management of disease affected the ability of spouses to provide support for patients	Belief about relationship roles

EAA, East Asian American; PCP, primary care provider.

### Cultural beliefs about food

Four studies included participant views on food. Beliefs about the role of food in health and the importance of having a traditional diet directly affected dietary self-management of T2DM.

Patients reported receiving dietary recommendations that did not align with their beliefs of food as medicine and a source of balance in life.^10,36^ They also expressed frustration because often a basic expression of care in times of illness is to provide food, especially ones that are viewed as beneficial to healing.^25^ One Chinese American patient was unhappy with this aspect of the management of diabetes, saying “others were always telling me to not eat … I have to live.”^24,36^ Similarly, Korean American patients found it difficult to adapt to recommended practices due to traditional views on food and that foods such as rice and meat were considered “good foods.” Another patient found that “the joy of living was gone after I knew that I had diabetes since I can't enjoy all the delicious [good] food anymore.”^24^

Patients reported that recommendations from health care providers typically involved removing refined carbohydrates from their diet, causing them to feel distanced from familiar and shared cultural food habits and practices. Chinese American patients noted that rice is a vital food, both physically and symbolically, and because of this, “the primary conflict is always the diet.”^10^ It was reportedly difficult to remove such a critical cultural staple from their diet.

### Social roles and food

Seven studies included participant views on social roles. The cultural views on social roles that influenced dietary self-management of T2DM were beliefs about spousal/relationship roles and beliefs about maintaining social harmony.

In EAA families with T2DM patients, dietary management and food preparation become a responsibility of the patient, as well as the spouse. Both Chinese American and Korean American patients reported that having the help and support of a spouse was critical to successful dietary self-management of diabetes.^25,26^

At the same time, diabetes may also put a significant strain on spousal relationships because of the expectation for support. Dietary modifications often affected both partners even if only one had diabetes.^10,25,26^ Spouses without diabetes who were reluctant to adapt the appropriate modifications expressed frustration, which ultimately ended in conflict centered on who should create, observe, and enforce food restrictions.^10^ This issue is often further complicated by traditional gender roles. Women generally prepare meals in the household; therefore, it can be difficult for EAA women with T2DM to manage various dietary preferences within the household, in addition to her own.^32^

The idea of not wanting to be a burden to others and not embarrassing oneself was consistent throughout the reviewed studies. Patients expressed their concern with being a burden in social settings where food was provided.^36^ They did not want hosts to feel obligated to accommodate the dietary modifications for management of diabetes, resulting in many EAA patients reluctant to tell others about their disease.^32,33^ Patients expressed concern that any dietary modification made would detract from the taste of the food^31^ and sense of cultural identity.^33^ Instead, they were more content with handling their dietary needs in a socially gracious manner and maintaining social harmony.^25^

### Culturally competent health services

Four studies included participant views on culturally competent health services. The views on culturally competent health services that influenced dietary self-management of T2DM were interactions with health care providers and bilingual health education resources.

Receiving bilingual and bicultural health care and health information was critical to successful dietary self-management of T2DM among EAAs. Culturally competent and bilingual medical staff with knowledge of culturally relevant foods enhanced diabetes management practices and were better able to address patient concerns.^35,36^ In contrast, patients who reported feeling disconnected from their physician did not feel their doctor always understood questions concerning diet or alternative food choices.^24^

In addition, because Cantonese, Mandarin, or Korean was the primary language for most EAA patients, diabetes education material in Chinese or Korean reportedly helped patients better understand precisely how to manage the disease.^28^ Patients stated that there was a lack of foreign language material that would help, for example, with knowing exactly how much rice was appropriate to eat.^35^ These issues were further compounded by lack of English proficiency among patients, which prevented them from receiving the most up-to-date and relevant diabetes information.^31^

### Characteristics of included quantitative studies

Six studies (*n*=347 participants)^27,29,30,34,37,38^ were reviewed for culturally tailored education interventions and their approaches used in dietary self-management of T2DM in EAAs (Table 3). Two studies used a randomized controlled trial design, one study used nonrandomized controlled cohort design, and three studies used a nonrandomized single-group cohort design. Three studies involved Chinese Americans and three studies involved Korean Americans. Sample size ranged from 23 to 92 participants with a mean age range from 56 to 70 years.

**Table 3.** Key Characteristics and Findings of Quantitative Studies on Diabetes Self-Management in East Asian Americans

Quantitative studies	Research design	Population	Setting	Intervention/control group	Cultural tailoring	Outcome measures	Major findings
Wang and Chan^34^	Nonrandomized single-group cohort study based on empowerment model	33 Chinese Americans (52% female), mean age 68.8±10.1 years	Community clinic in Hawaii	10 weekly group education sessions with certified diabetes instructor/not applicable	Education integrating Chinese language, dietary examples, exercise suggestions, traditional medicine, and cultural beliefs into plans for self-management of disease with diet, exercise, medication, and self-care	Quality of life (modified DQOL), HbA1c levels, body weight, BP	Decreased mean HbA1c of 0.99% (*p*-value not reported, *d*=0.53, 95% CI [0.03–1.01]) and increased diabetes self-management knowledge at 3 months. Language of instruction and dietary suggestions were important features of the intervention
Kim et al.^30^	Randomized controlled trial based on CBPR	79 Korean Americans (44% female), mean age 56.5±7.9 years	Korean Resource Center in Washington DC/Baltimore Area	6 weekly group education sessions, followed by home glucose monitoring and individual monthly telephone counseling with bilingual nurse for 24 weeks/usual care	Education and counseling by trained bilingual nurses about diabetes, management, complications, healthy eating, culturally relevant food suggestions food labels, exercise, medications, and communicating with physician	Diabetes knowledge (DKT), Self-efficacy (SCDSE), Diabetes self-care activities (SDSCA), Depression (KDSKA), Quality of life (DQOL), HbA1c, fasting glucose	Decreased mean HbA1c of 0.9% (*p*=0.01, *d*=0.67, 95% CI [0.21–1.11]), fasting glucose 35.3 mg/dL (*p*=0.06). Increased diabetes knowledge (*p*<0.01), quality of life (*p*=0.03), self-care activities (*p*<0.01), and self-efficacy (*p*=0.01) at 30 weeks
Song et al.^37^	Randomized controlled trial based on CBPR	79 Korean Americans (44% female), mean age 56.5±7.9 years	Korean Resource Center in Washington DC/Baltimore Area	6 weekly group education and interactive sessions led by bilingual instructor/usual care	Education available in preferred language. Individually tailored serving tables and culture-specific food model with considerations for Korean-specific diet and food preparation	Diabetes knowledge (DKT), satisfaction survey with self-designed open-ended question survey	Increased diabetes-related nutrition knowledge (*p*<0.01), mean satisfaction of 2.7 out of 3 on educational content at 30 weeks
Ivey et al.^29^	Nonrandomized controlled cohort study based on Bodenheimer model	92 Chinese Americans (65% female), mean age 66.7±10.7 years	Asian Health Services in Oakland, CA	3 individual visits with physician and registered dietitian and follow-up calls with a health coach over a period of 6 months/usual care	Education by trained medical assistants with Chinese language diabetes education materials. Dieticians and physicians linguistically matched. Recommendations culture sensitive	HbA1c levels	Decreased mean difference of HbA1c 0.36% (*p*=0.14, *d*=0.28, 95% CI [−0.14 to 0.68]) at 8.5 months. Health coach addressed psychosocial factors affecting self-management such as navigating the health system and providing moral support
Choi and Rush^27^	Nonrandomized single-group cohort study	41 Korean Americans (54% female), mean age 70.3±8.4 years	Community center on the West Coast	2 group education sessions lead by a bilingual family nurse practitioner/not applicable	Education lead by bilingual family nurse practitioners with cultural tailoring employing native language, using cultural dietary preferences, and discussions of cultural beliefs in relationship to treatment and practices	Self-management (SDSCA), Diabetes knowledge (DKT), mood (PHQ-9), Health status (SF-12), HbA1c, BMI	Decreased mean HbA1c of 0.52% (*p*<0.001, *d*=0.36, 95% CI [0.07–0.80]), increased feet checks 1.4 times per week (*p*<0.001), increased diabetes knowledge (*p*=0.39), increased diabetes self-efficacy (*p*=0.098) at 3 months
Sun et al.^38^	Nonrandomized single-group cohort study based on CCM, TRA, and SCT	23 Chinese Americans (52% female), 52% age 70–79, 22% age 80–89	Medical office building in San Francisco	12 group support sessions lead by multidisciplinary bilingual team over 6 months and bilingual booklet on diabetes management/not applicable	Education lead by bilingual team of registered nurses, dietitians, and CDEs incorporated Chinese commonly practiced activities and culturally relevant foods into curriculum. Discussed use of traditional medicine and exercise	Diabetes knowledge (based off ADA recommendations), diabetes care activities (self-report questionnaire), and HbA1c	Decreased mean HbA1c of 0.76% (*p*=0.001, *d*=0.93, 95% CI [0.31–1.53]) and increased diabetes knowledge (*p*<0.01) at 6 months. Moral support in group setting helped to improve self-management

ADA, American Diabetes Association; BMI, body mass index; CBPR, community-based participatory research; CCM, chronic care model; CDE, certified diabetes educator; CI, confidence interval; DKT, diabetes knowledge test; DQOL, diabetes quality-of-life measure; KDSKA, Kim Depression scale for Korean Americans; N/A, not available; PHQ-9, Patient Health Questionnaire; SCDSE, Stanford Chronic Disease Self-Efficacy scale; SCT, social cognitive theory; SDSCA, summary of diabetes self-care activities; SF-12, Abbreviated Medical Outcomes SF-36 Health survey; TRA, theory of reasoned action.

### Synthesis of quantitative review findings

Two studies incorporated individual, one-on-one education sessions and four studies used group education sessions. The most commonly incorporated cultural approaches in the diabetes education were bilingual education (100% of interventions) and culturally specific dietary recommendations (100% of interventions). Half of the interventions incorporated other EAA cultural considerations such as specific exercise recommendations and considerations about traditional medicine as it relates to T2DM management.

The most common outcome measures used in the six studies were HbA1c levels, followed by diabetes knowledge, and diabetes self-care behaviors. Among the studies assessing the intervention effects on HbA1c, 100% reported absolute reduction in HbA1c and 60% reported statistically significant (*p*<0.05) reduction in HbA1c from baseline to follow-up, ranging from 3- to 8.5-month duration.^29,30,34,37,38^ There was consistent evidence of a positive effect of the interventions on HbA1c (Table 3). Of studies assessing diabetes knowledge, the majority reported a significant increase,^30,34,37,38^ and of studies assessing self-care behaviors, 33% reported a significant increase.^27,30^

### Integrated synthesis of qualitative and quantitative review findings

Analysis of qualitative studies revealed that cultural beliefs about food, social roles and food, and culturally competent health services should be considered when addressing dietary self-management of T2DM among EAAs. When examining whether these concepts were in any way built in to the approaches used in the education interventions, we found that cultural beliefs about food and offering culturally competent health services were consistently addressed in the quantitative studies. All six studies directly addressed cultural beliefs about food by teaching about culturally sensitive dietary modifications and offering appropriate suggestions for substitutes, and bilingual instructors were provided as a form of culturally competent health service in all of the education interventions. Tools to help improve communication about diabetes care to others, as well as the social impact of managing diabetes were not explicitly mentioned in any of the studies.

## Discussion

This mixed-methods systematic review examined dietary self-management of T2DM in EAAs and the cultural factors influencing management with the goal of providing direction for interventions to improve patient self-management and thereby, outcomes. Based on the qualitative and quantitative studies reviewed, it was evident that the following cultural influences play an important role in dietary self-management of T2DM among EAAs:

(1) Beliefs about food, including beliefs about food as medicine and beliefs about traditional East Asian diets
(2) Beliefs about social roles, including the spousal relationship and maintaining social harmony
(3) Receiving bilingual and bicultural health care and health education

For EAA patients, culturally rooted views on food can be deeply ingrained. High glycemic index foods such as rice are viewed as healthy and to an extent, medicinal^10,25^; therefore, being restricted from consuming certain foods can be counterintuitive when interpreting dietary recommendations. In the context of social relationships, harmony at home and in the community can be affected by diabetes self-management. The spousal relationship is often immediately affected by changes in an EAA patient's diet conducive to diabetes management.^10,25,26^ Patients may also perceive their diet as a burden to others in the social setting^32,33^ and understandably, are reluctant to adapt large changes from their traditional diet. These concerns need to be addressed in the self-management of the patients' diabetes.

This review highlighted several important considerations in self-management education for EAA patients with T2DM. Interventions offered specific recommendations for diet modification, such as recipes, providing bilingual resources for health education, and delivered care from bilingual and bicultural providers. In their 2017 update to standards for diabetes self-management education,^41^ the American Diabetes Association marks “individualization” as a standard component of education. More specifically, cultural influences, health beliefs and attitudes, peer support, and literacy level are identified as some of the factors affecting individualized care.^41–44^ The success of these education interventions at reducing HbA1c and increasing diabetes knowledge reflects the value of offering culturally appropriate dietary suggestions and bilingual education materials.

In addition, the utility of group-based education sessions was evident in the results of the interventions. It is well-documented that group-based diabetes self-management education in people with T2DM leads to improvements in clinical, lifestyle, and psychosocial outcomes.^45^ Most interventions in the review^27,30,34,37,38^ held education sessions in group settings on a regular basis, providing a safe environment for patients to learn about their disease and share experiences with sympathetic peers. Other intervention features that may have been important to producing positive outcomes were the number of sessions, duration of follow-up, and type of diabetes educator, although no clear association could be made between these features and significant decrease in HbA1c or increase in diabetes knowledge. Notably, the one study^29^ that did not report significant impact on HbA1c involved three individual visits with a physician and registered dietitian and follow-up phone calls with a health coach over a 6-month period and no group-based education.

Several limitations of this review should be acknowledged. Differing levels of acculturation, referring to the changes that take place as a result of contact with culturally dissimilar people, groups, and social influences,^46^ may have resulted in different patient views on the importance of cultural accommodations in the management of diabetes. More acculturated immigrants are more likely to consume American foods,^18,47,48^ which may have affected their responses in qualitative studies or their reception to suggestions in the quantitative study interventions.

Quantitative trial design and sample diversity were also limitations. Three of the six quantitative studies used nonrandomized single-group cohort design rather than the more rigorous randomized controlled trial, lowering the validity of their findings. In addition, no studies included Japanese American immigrants, possibly because they are a smaller immigrant population and have fewer enclaves of new immigrants than their Chinese and Korean counterparts. Geographic location of the study populations may also have affected results, as only areas with dense, urban populations of EAA were studied. Last, although there are many cultural similarities among EAAs, specific health beliefs may differ.

As the EAA population grows, there is a greater need for health care providers to be aware of the relevant considerations that need to be made in treating EAA patients with diabetes. The differences between a traditional East Asian and Western diet and cultural understanding of diabetes must be considered when making recommendations to EAA patients. The outcomes of this mixed-methods systematic review provide a useful starting point from which to continue to develop and modify culturally tailored education interventions to improve dietary self-management of T2DM among EAAs. Future study should be aimed at further elucidating particular interventions that are effective at producing positive diabetes-related outcomes in a diverse range of EAA populations with different levels of acculturation and in different geographic locations.

## Abbreviations Used

ADA: American Diabetes Association
BMI: body mass index
CBPR: community based participatory research
CCM: chronic care model
CDE: certified diabetes educator
CI: confidence interval
DKT: diabetes knowledge test
DQOL: diabetes quality of life measure
EAA: East Asian American
HbA1C: hemoglobin A1c
KDSKA: Kim Depression scale for Korean Americans
N/A: not available
NHANES: National Health and Nutrition Examination Survey
PCP: primary care provider
PHQ-9: Patient Health Questionnaire
PRISMA: Preferred Reporting Items for Systematic Review and Meta-Analysis
SCDSE: Stanford Chronic Disease Self-Efficacy scale
SCT: social cognitive theory
SDSCA: summary of diabetes self-care activities
SF-12: Abbreviated Medical Outcomes SF-36 Health survey
T2DM: type 2 diabetes mellitus
TRA: theory of reasoned action
